# Specific Egg Yolk Immunoglobulin as a New Preventive Approach for Shiga-Toxin-Mediated Diseases

**DOI:** 10.1371/journal.pone.0026526

**Published:** 2011-10-19

**Authors:** Paola Neri, Shunji Tokoro, Ryo Kobayashi, Tsuyoshi Sugiyama, Kouji Umeda, Takeshi Shimizu, Takao Tsuji, Yoshikatsu Kodama, Keiji Oguma, Hiroshi Mori

**Affiliations:** 1 Microbiology, Department of Biopharmaceutical Sciences, Gifu Pharmaceutical University, Gifu, Japan; 2 Immunology Research Institute, GHEN Corporation, Gifu, Japan; 3 Department of Molecular Infectiology, Graduate School of Medicine, Chiba University, Chiba, Japan; 4 Department of Microbiology, School of Medicine, Fujita Health University, Aichi, Japan; 5 Department of Bacteriology, Graduate School of Medicine, Dentistry and Pharmaceutical Sciences, Okayama University, Okayama, Japan; Institut de Pharmacologie et de Biologie Structurale, France

## Abstract

Shiga toxins (Stxs) are involved in the development of severe systemic complications associated with enterohemorrhagic *Escherichia coli* (EHEC) infection. Various neutralizing agents against Stxs are under investigation for management of EHEC infection. In this study, we immunized chickens with formalin-inactivated Stx-1 or Stx-2, and obtained immunoglobulin Y (IgY) from the egg yolk. Anti-Stx-1 IgY and anti-Stx-2 IgY recognized the corresponding Stx A subunit and polymeric but not monomeric B subunit. Anti-Stx-1 IgY and anti-Stx-2 IgY suppressed the cytotoxicity of Stx-1 and Stx-2 to HeLa 229 cells, without cross-suppressive activity. The suppressive activity of these IgY was abrogated by pre-incubation with the corresponding recombinant B subunit, which suggests that the antibodies directed to the polymeric B subunits were predominantly involved in the suppression. *In vivo*, the intraperitoneal or intravenous administration of these IgY rescued mice from death caused by intraperitoneal injection of the corresponding toxin at a lethal dose. Moreover, oral administration of anti-Stx-2 IgY reduced the mortality of mice infected intestinally with EHEC O157:H7. Our results therefore suggest that anti-Stx IgY antibodies may be considered as preventive agents for Stx-mediated diseases in EHEC infection.

## Introduction

Enterohemorrhagic *Escherichia coli* (EHEC) causes a spectrum of human diseases, including diarrhea, hemorrhagic colitis, disordered consciousness, renal failure and hemolytic uremic syndrome (HUS) [1], [2]. EHEC colonizes the large intestine and produces Shiga toxins (Stxs) as major virulence factors that are involved in the pathogenicity of EHEC infections. EHEC produces two types of Stxs, Stx-1 and Stx-2. Stxs released into the intestinal lumen enter the systemic circulation and reach target organs, where they manifest their toxicity [3], [4]. Stxs are multimeric proteins that consist of an A subunit (32 kDa) and a pentamer of identical B subunits (7.7 kDa each). The A subunit links non-covalently to the pentamer of B subunits. The B pentamer is responsible for the toxin binding to target cell-surface glycolipid receptors such as galabiosyl ceramide (Gb_2_-Cer) and globotriaosyl ceramide (Gb_3_-Cer) [5], [6]. After binding, the toxin is internalized into the cells, and the A subunit is cleaved into A1 (28 kDa) and A2 (4 kDa) fragments. The A1 fragment exerts RNA *N*-glycosidase activity, which results in inhibition of protein synthesis by inactivation of 28S rRNA [7], [8].

The treatment of EHEC infection with antibiotics is controversial because of the increased risk of HUS owing to enhancement of Stx induction/release [9]. It has been reported that some antibiotics markedly enhance Stx-2 production from EHEC by inducing Stx-encoding bacteriophages [10], and release Stx-1 that is stored in the periplasm of EHEC in large amounts [11]. Based on these findings, alternative methods for therapy or prevention of Stx-mediated diseases have been investigated worldwide.

Various synthetic compounds that mimic natural receptors, Gb_2_-Cer or Gb_3_-Cer, have been studied for eliminating Stxs from the intestine by binding to and/or neutralizing Stxs in the circulation as a therapeutic strategy for protecting patients from serious Stx-mediated diseases [12]–[18]. Clinically, Stx-2 is more potent in the development of Stx-mediated diseases than Stx-1 [19], [20]. In mice, the lethal dose of Stx-2 is 400 times lower compared with Stx-1 [21]. In contrast, it has been shown that the affinity of Stx-1 for the glycolipid receptor is higher than that of Stx-2 [22], [23]. Therefore, the Gb_2_- or Gb_3_-conjugated compounds that have been reported as drug candidates show a good neutralizing activity against Stx-1, but are less active against Stx-2, although we recently reported that Gb_2_/Gb_3_-conjugated to phosphatidyl residues is relatively effective at neutralizing Stx-2 [18]. Importantly, it has been speculated that the low affinity of Stx-2 for the receptor might be involved in the higher toxicity *in vivo* [22]. A potential approach for the treatment of Stx-mediated diseases may therefore be the development of agents that bind Stx-2 with high affinity, and thereby preventing the binding of Stx-2 to its natural receptor.

Beside the development of compounds that mimic Stx-2 receptors, monoclonal antibodies against Stxs have also been investigated for the prevention of Stx-mediated diseases [24]–[27]. Compared with the synthetic compounds, antibodies are expected to neutralize Stx-2 effectively, owing to a different neutralizing mechanism. Antibodies have substantially higher molecular weight than the intact toxin. The antibodies might interfere with the binding of Stx to the receptor through steric hindrance and probably structural instability, regardless of the epitope recognized. This is vastly different from the inhibition mechanism of the synthetic compounds that are required to bind to the B subunit binding site. Antibodies are usually administered systemically through parenteral routes. However, EHEC produces Stx in the intestine, from where the toxin enters the bloodstream to reach the target organ [3], [4]. While we believe that neutralization of Stxs in the intestine by oral treatment is more desirable than neutralization in the vessel by i.v. treatment for preventing Stx-mediated diseases, oral administration requires greater quantities of antibodies compared with parenteral administration.

Recently, there has been increasing interest in the oral administration of antibodies for localized treatment of gastrointestinal infections [28]. Chicken egg yolk has been recognized as an economical source of polyclonal antibody for oral administration [29], and egg yolk immunoglobulin Y (IgY) of chickens immunized with pathogenic microbes has been extensively studied for the treatment of various gastroenteric infectious diseases [30]–[34].

In the present study, we focused on IgY antibodies against Stxs for oral use to prevent Stx-mediated diseases. Previous investigations using monoclonal and polyclonal antibodies against Stxs demonstrated that anti-Stx-1 antibodies do not cross-neutralize Stx-2, and anti-Stx-2 antibodies do not neutralize Stx-1 [35], [36], although the A subunits of Stx-1 and Stx-2 have 55% and B subunits of Stx-1 and Stx-2 have 57% deduced amino acid sequence homology [37]. We therefore immunized chickens with formalin-inactivated Stx-1 and Stx-2 individually, and characterized the obtained IgY preparations for antibody activity that may potentially allow the prevention of Stx-mediated diseases.

## Results

### IgY preparation of chicken immunized with partially purified Stx-1 and Stx-2

Stx-1 and Stx-2 were prepared using bacterial strains (Table 1), and partially purified preparations (ppStx-1 and ppStx-2) were used for immunization of chickens, as described in the Materials and Methods. Fifty-three eggs were collected from chickens immunized with Stx-1, and 50 eggs from Stx-2-immunized chickens. Egg yolks were pooled, purified by ammonium sulfate precipitation and finally freeze-dried. Preparations from Stx-1- and Stx-2-immunized chickens are referred to as anti-Stx-1 IgY and anti-Stx-2 IgY, respectively (Table 2).

**Table 1:** EHEC or *E. coli* strains used in this study.

Used for toxin preparation				
Strain^a^	Producing	Purification	Referred as	Used in
GPU96MM	Stx-1 and Stx-2			
BL21/pETSTX1	Recombinant Stx-1	partially	ppStx-1^b^	Tables 3 and 4, Figure 5
		highly	hpStx-1	Figures 1–4
GPU993	Stx-2	partially	ppStx-2^c^	Tables 3 and 4, Figure 5
		highly	hpStx-2	Figures 1–4
JM109/p2Ais-10	Hybrid toxin of Stx-2 A subunit and Stx-1 B subunit	highly	hpStx-2A1B	Figure 2

Used for infection experiments			
Streptomycin resistant strain	Producing	Original strain	Used in
GPU96MM-S	Stx-1 and Stx-2	GPU96MM	Figure 6
GPU993-S	Stx-2	GPU993	

a see “bacterial strains” in Materials and Methods for detail.

b ppStx-1 and

c ppStx-2 were used for *in vivo* experiments that required a large amount of Stxs.

**Table 2:** Anti-Stx-1 IgY and anti-Stx-2 IgY preparations.

	Anti-Stx-1 IgY	Anti-Stx-2 IgY
Number of eggs	53	50
Total yolk weight (g)	924	846
Freeze-dried powder (g)	8.2	8.6
Protein concentration (g/g powder)^a^	0.930	0.970
IgY concentration (g/g protein)^b^	0.148	0.230

a assayed using DC™ Protein assay (BioRad, Hercules, CA, USA).

b assayed using Chicken IgG ELISA Quantitation Kit™ (Bethyl Laboratories, Inc., Montgomery, TX, USA).

### Western blotting and dot blot analysis of anti-Stx-1 IgY and anti-Stx-2 IgY

**Figure 1:**
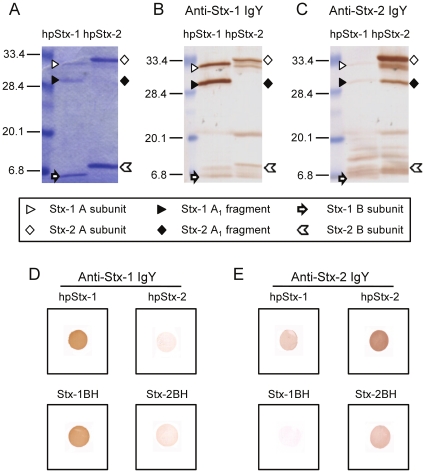
Western blot analysis and dot blot analysis. (A–C) Western blot analysis of anti-Stx-1 IgY and anti-Stx-2 IgY. hpStx-1 and hpStx-2 were subjected to SDS-PAGE at 2.5 µg/lane. (A) CBB staining of SDS-PAGE gel. (B) Anti-Stx-1 IgY and (C) anti-Stx-2 IgY were applied to the membrane at 20 µg/ml. The marks in the figures indicate A subunit, A1 fragment and monomeric B subunit. (D, E) Dot blot analysis of anti-Stx-1 IgY and anti-Stx-2 IgY. hpStx-1 and hpStx-2 were blotted at 4 µg/dot, and Stx-1BH and Stx-2BH at 2 µg/dot. Anti-Stx-1 IgY and anti-Stx-2 IgY were applied to the membrane at 2 µg/ml and 4 µg/ml, respectively.

We initially assessed the specificity of anti-Stx-1 IgY and anti-Stx-2 IgY by western blotting. Highly purified Stx-1 (hpStx-1) and Stx-2 (hpStx-2) were subjected to SDS-PAGE (Table 1 for bacterial strains used for toxin preparation). Coomassie Brilliant Blue (CBB) staining of SDS-PAGE gels loaded with hpStx-1 revealed bands at 32.2 kDa and 6.1 kDa, indicating the Stx-1 A subunit and monomeric B subunit, respectively. The staining intensity of the B subunit was stronger than the A subunit (Figure 1A). For hpStx-2, bands at 33.1 kDa and 8.8 kDa indicated the Stx-2 A subunit and monomeric B subunit, respectively. Similarly to hpStx-1, the staining intensity of the B subunit was stronger than the A subunit. Bands of hpStx-1 and hpStx-2 at 28.6 kDa most likely represent A1 fragments. The staining intensity of the Stx-1 A1 fragment was stronger compared with that of the Stx-1 A subunit. In contrast, the staining intensity of the Stx-2 A1 fragment was weaker than that of the Stx-2 A subunit. Western blots of these samples were then probed with anti-Stx-1 IgY and anti-Stx-2 IgY (Figure 1B, C). Anti-Stx-1 IgY produced clear bands at the expected positions of the Stx-1 A subunit and the A1 fragment, compared with Stx-2 (Figure 1B). In contrast, anti-Stx-2 IgY produced clear bands at the expected positions of the Stx-2 A subunit and the A1 fragment, compared with Stx-1 (Figure 1C). Anti-Stx-1 IgY and anti-Stx-2 IgY produced only faint bands at the expected positions of Stx-1 and Stx-2 B subunits, respectively (Figure 1B, C).

We further assessed the specificity of anti-Stx-1 IgY and anti-Stx-2 IgY by dot blot analysis. As shown in Figure 1D, anti-Stx-1 IgY reacted strongly with hpStx-1 and recombinant Stx-1 B subunit tagged with histidine (Stx-1BH), but weakly with hpStx-2 and recombinant Stx-2 B subunit tagged with histidine (Stx-2BH). Similarly, anti-Stx-2 IgY reacted strongly with hpStx-2 and Stx2-BH, but weakly with hpStx-1 and Stx-1BH (Figure 1E).

### Neutralizing activity of anti-Stx-1 IgY and anti-Stx-2 IgY against Stx

**Figure 2 pone-0026526-g002:**
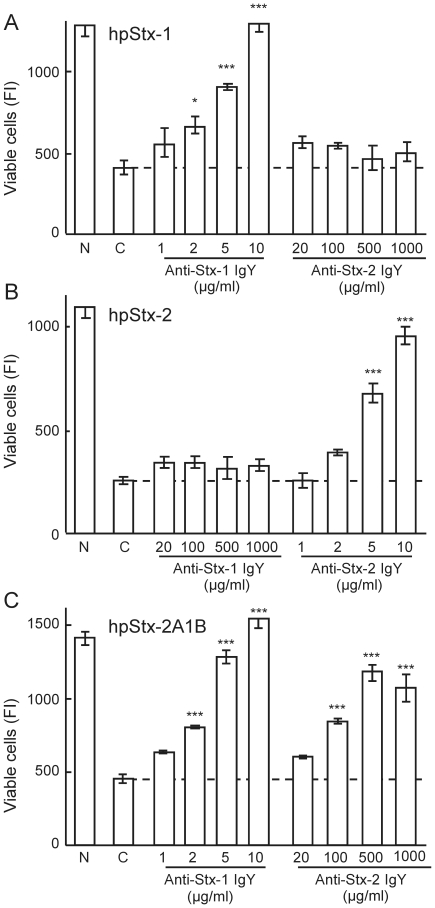
Neutralizing activity of anti-Stx IgY. (A) hpStx-1, (B) hpStx-2 and (C) hpStx-2A1B were mixed with serial dilutions of anti-Stx-1 IgY and anti-Stx-2 IgY, and incubated for 1 h at 37°C. The mixture was subjected to the cytotoxicity assay. Final concentration of hpStx-1, hpStx-2 and hpStx-2A1B was five times CD_50_. N: cells not treated with Stx. C: cells treated with Stx alone (control). Each column represents the mean ± SEM of triplicate wells. There was a significant difference at *p<0.05, **p<0.01 and ***p<0.001 vs. the control (Dunnet's comparison test).

The neutralizing activity of the IgY was assessed against five times the 50% cytotoxic dose (CD_50_) of hpStx-1, hpStx-2 and purified hybrid toxin composed of A subunit of Stx-2 and B subunit of Stx-1 (hpStx-2A1B) (Figure 2). IgY was preincubated with the different Stx preparations and subsequently subjected to the cytotoxicity assay. Anti-Stx-1 IgY neutralized hpStx-1 in a concentration-dependent manner, with a 50% neutralizing concentration (NC_50_) of 3.4 µg/ml (95% confidence interval (CI): 2.5–4.5 µg/ml), while anti-Stx-2 IgY had no effect, even at the highest concentration of 1000 µg/ml (Figure 2A). Similarly, anti-Stx-2 IgY neutralized hpStx-2 with an NC_50_ of 4.8 µg/ml (95% CI: 4.2–5.5 µg/ml), while anti-Stx-1 IgY had no effect (Figure 2B). Anti-Stx-1 IgY and anti-Stx-2 IgY neutralized hpStx-2A1B with an NC_50_ of 2.7 µg/ml (95% CI: 2.4–3.1 µg/ml), and 113.3 µg/ml (95% CI: 73.4–174.9 µg/ml), respectively (Figure 2C). The neutralizing activity of anti-Stx-1 IgY was similar for hpStx-2A1B and hpStx-1. Anti-Stx-2 IgY, however, did not show complete neutralization, even at 1000 µg/ml.

### Effect of Stx-1BH and Stx-2BH on the neutralizing activity of anti-Stx-1 IgY and anti-Stx-2 IgY against Stx

**Figure 3 pone-0026526-g003:**
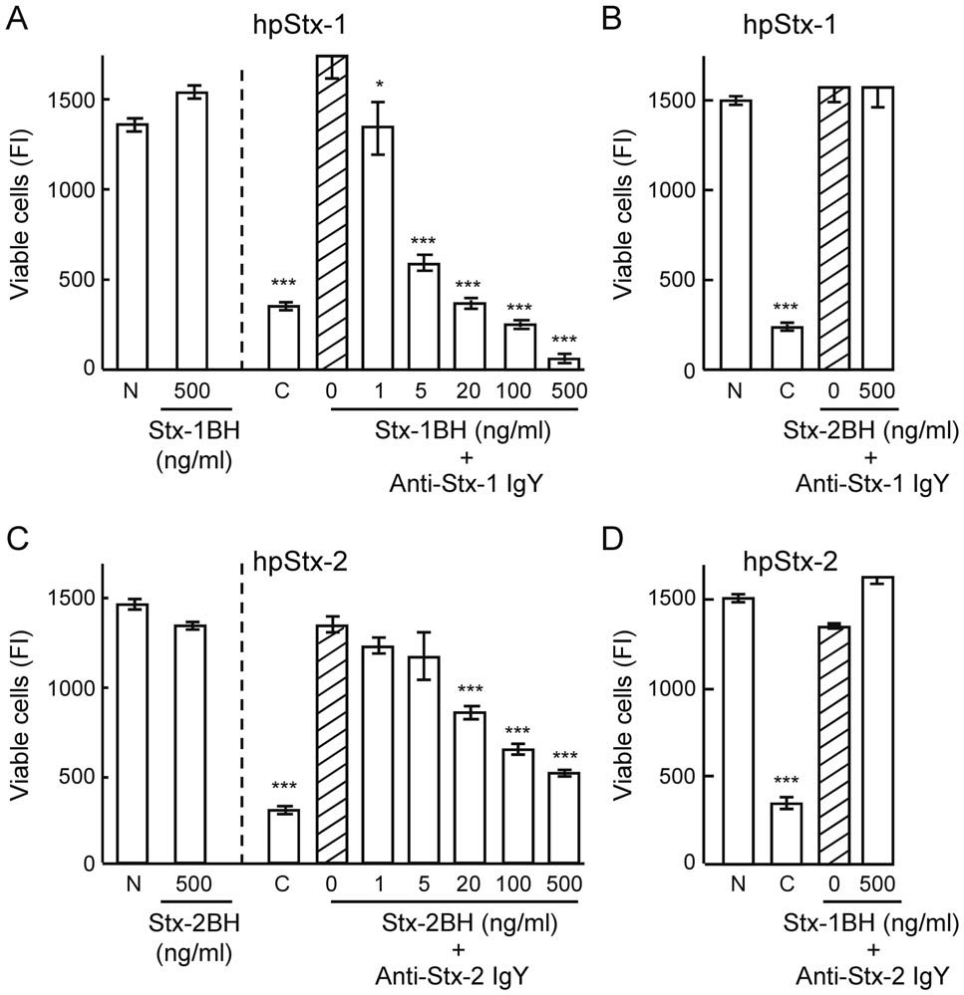
Effect of Stx B subunit on the neutralizing activity of anti-Stx IgY. (A, B) Anti-Stx-1 IgY and (C, D) anti-Stx-2 IgY were mixed with serial dilutions of Stx-1BH and Stx-2BH, and incubated for 2 h at 37°C. (A, B) hpStx-1 and (C, D) hpStx-2 were added to the mixture, and incubated at 37°C for a further 1 h. The resultant mixture was subjected to the cytotoxicity assay. Final concentrations of hpStx-1 and hpStx-2 were five times CD_50_, and those of anti-Stx-1 IgY and anti-Stx-2 IgY were 10 µg/ml. N: cells not treated with Stx. C: cells treated with Stx alone (control). Hatched column (0): cells treated with Stx and anti-Stx IgY without B subunit. Each column represents the mean ± SEM of triplicate wells. There was a significant difference at *p<0.05, **p<0.01 and ***p<0.001 vs. the hatched column (Dunnet's comparison test).

The neutralizing activity of anti-Stx-1 IgY and anti-Stx-2 IgY were assessed after preincubation with Stx-1BH and/or Stx-2BH. Stx-1BH decreased the neutralizing activity of anti-Stx-1 IgY against hpStx-1 in a concentration-dependent manner (Figure 3A), while Stx-2BH had no effect, even at the highest concentration (Figure 3B). On the contrary, Stx-2BH, but not Stx-1BH, decreased the activity of anti-Stx-2 IgY against hpStx-2 in a concentration-dependent manner (Figure 3C, D).

### Neutralizing activity of anti-Stx-1 IgY and anti-Stx-2 IgY on Stx-1 and Stx-2 bound to target cells

**Figure 4:**
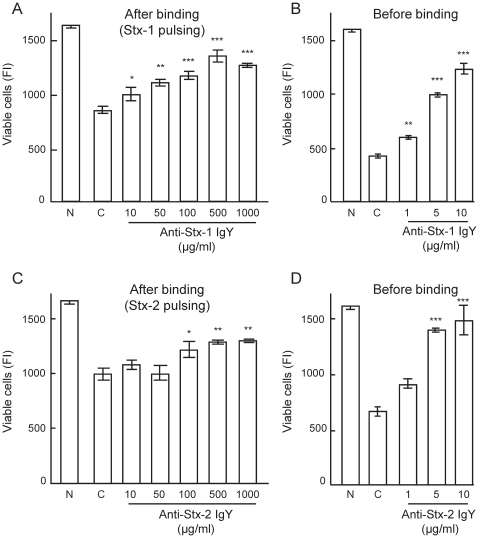
Neutralizing activity of anti-Stx IgY on Stx bound to target cells. (A) hpStx-1 and (C) hpStx-2 were added to HeLa 229 cells at a concentration of 20 times CD_50_, and kept on ice for 30 min. Cells were extensively washed with cold DMEM to remove unbound Stx. (A) Anti-Stx-1 IgY and (C) anti-Stx-2 IgY were added to the Stx-pulsed cells and the cytotoxicity assay was performed. (B) Anti-Stx-1 IgY and (D) anti-Stx-2 IgY were added to HeLa 229 cells just before addition of (B) hpStx-1 and (D) hpStx-2 at a concentration of five times CD_50_, and the cytotoxicity assay was performed. N: cells not treated with Stx. C: cells treated with Stx alone (control). Each column represents the mean ± SEM of quadruplicate wells. There was a significant difference at *p<0.05, **p<0.01 and ***p<0.001 vs. the control (Dunnet's comparison test).

hpStx-1 and hpStx-2 were loaded on to HeLa 229 cells in a pulsing manner, as described in the legend for Figure 4. The pulsing of cells with hpStx-1 and hpStx-2 alone (control) showed 52% and 59% cytotoxicity, respectively (Figure 4A, C). When anti-Stx-1 IgY and anti-Stx-2 IgY were added to the pulsed cells, only partial neutralization of the toxins was detected, even at the highest concentration of 1000 µg/ml. On the other hand, when anti-Stx-1 IgY and anti-Stx-2 IgY were added to the cells before hpStx-1 and hpStx-2 loading, efficient neutralization of the toxins was measured, with NC_50_ values of 5.2 µg/ml (95% CI: 2.8–9.7 µg/ml) and 2.1 µg/ml (95% CI: 0.9–4.9 µg/ml), respectively (Figure 4B, D).

### Effect of anti-Stx-1 IgY and anti-Stx-2 IgY on mortality of mice challenged i.p. with a lethal dose of Stx-1 and Stx-2

ppStx-1 and ppStx-2 were used for the mortality assay. After i.p. challenge with 2.5 times 50% lethal dose (LD_50_) ppStx-1 and two times LD_50_ ppStx-2, five out of six mice died by day 5 in the control group (Table 3). When anti-Stx-1 IgY was given i.p. at a dose of 100 mg/kg, 10 min before challenge with ppStx-1, none of the six mice died. After treatment with 10 mg/kg anti-Stx-2 IgY, two out of six mice challenged with ppStx-2 died, but at doses of 25 and 100 mg/kg, no mice died.

**Table 3:** Effect of anti-Stx-1 IgY and anti-Stx-2 IgY on mortality of mice challenged with ppStx-1 and ppStx-2.

ppStx-1		ppStx-2	
Anti-Stx-1 IgY (mg/kg)	Number of mice (Dead/Total)	Anti-Stx-2 IgY (mg/kg)	Number of mice (Dead/Total)
Control	5/6	Control	5/6
2.5	6/6	2.5	6/6
10	6/6	10	2/6
25	6/6	25	0/6***
100	0/6***	100	0/6***

ddY mice were administered i.p. with anti-Stx-1 IgY or anti-Stx-2 IgY, followed 10 min later by an i.p. challenge with ppStx-1 (2.5 times LD_50_) or ppStx-2 (2 times LD_50_). Death of mice was observed for 10 days after the challenges.

***There is a statistical significant difference at *p*<0.001 vs. the control (log rank test).

**Figure 5:**
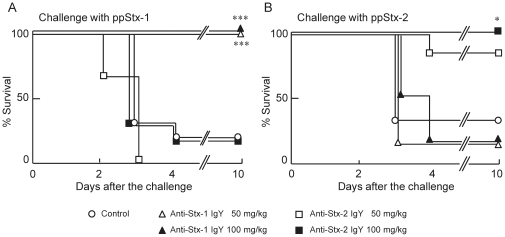
Effect of anti-Stx IgY given via different routes from Stx on mortality of mice. (A) ppStx-1 (2.5 times LD_50_) and (B) ppStx-2 (2 times LD_50_) were administered i.p. to ddY mice. Anti-Stx-1 IgY and anti-Stx-2 IgY were given i.v. at doses of 50 and 100 mg/kg at 10 min before challenge. Control mice were given PBS instead of anti-Stx IgY. Each group included six mice. There was a significant difference at *p<0.05 and ***p<0.001 vs. the control (log rank test).

The protective efficacy was evaluated by i.v. injection of anti-Stx-1 IgY and anti-Stx-2 IgY (Figure 5). In mice challenged i.p. with ppStx-1 (Figure 5A), five out of six died in the control group. However, none of six mice treated with 50 or 100 mg/kg anti-Stx-1 IgY died. In contrast, all six mice treated with 50 mg/kg anti-Stx-2 IgY died, and five out of six mice treated with 100 mg/kg died. In mice challenged i.p. with ppStx-2 (Figure 5B), four out of six mice died in the control group. In mice treated with 50 mg/kg anti-Stx-2 IgY, one out of six died, and none of those treated with 100 mg/kg anti-Stx-2 IgY died. In contrast, five out of six mice died in each group treated with 50 and 100 mg/kg anti-Stx-1 IgY.

Time dependency of treatment with anti-Stx-1 IgY and anti-Stx-2 IgY was examined (Table 4). In the control group, five out of six mice challenged i.p. with ppStx-1 died by day 4. In contrast, none of six mice died that were treated i.v. with anti-Stx-1 IgY at 10 min before or simultaneously with ppStx-1 challenge. However, all six mice died when anti-Stx-1 IgY was given later than 10 min after the challenge. After i.p. challenge with ppStx-2, all six mice died in the control group, but none died when treated i.v. with anti-Stx-2 IgY 10 min before or simultaneously with ppStx-2 challenge. However, all mice died when anti-Stx-2 IgY was given later than 10 min after the challenge.

**Table 4:** Mortality of mice administered anti-Stx-1 IgY and anti-Stx-2 IgY after challenge with ppStx-1 and ppStx-2.

	Mortality (Dead/Total)	
	Anti-Stx-1 IgY	Anti-Stx-2 IgY
Timing of IgY administration (min)	ppStx-1	ppStx-2
Control	5/6	6/6
−10	0/6***	0/6***
0	0/6***	0/6***
10	6/6	6/6
20	6/6	6/6
30	6/6	6/6
60	6/6	6/6
120	6/6	6/6
360	6/6	6/6

ddY mice were challenged i.p. with 2.5 times LD_50_ ppStx-1 and 2 times LD_50_ ppStx-2. Anti-Stx-1 IgY and anti-Stx-2 IgY were given to mice i.v. at a dose of 100 mg/kg at various timing on the challenge.

***There is a statistical significant difference at *p*<0.001 vs. the control (log rank test).

### Effect of anti-Stx-1 IgY and anti-Stx-2 IgY on mortality of mice infected intestinally with GPU993-S and GPU96MM-S

**Figure 6:**
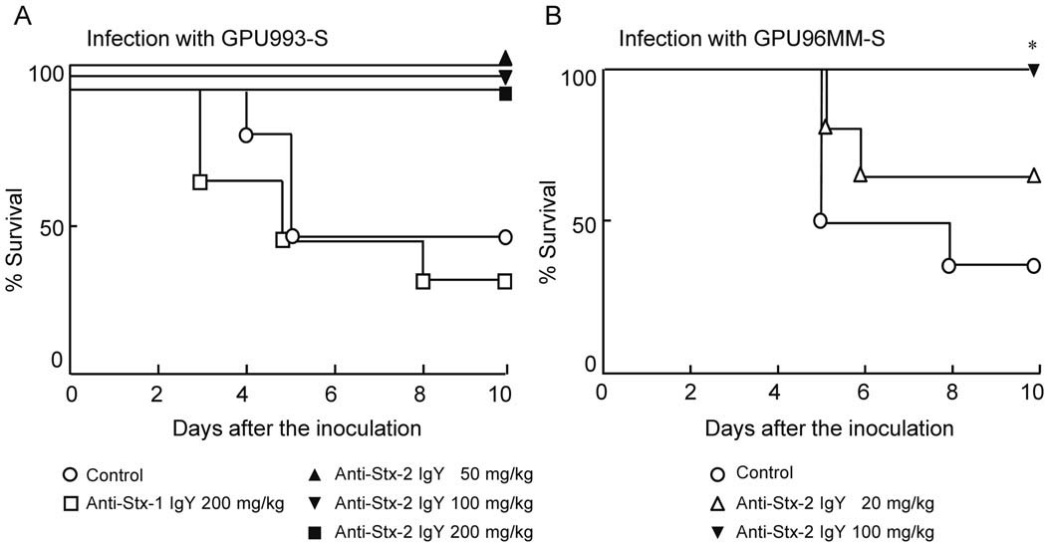
Effect of orally administered anti-Stx IgY on mortality of mice infected with EHEC O157:H7. (A) GPU993-S and (B) GPU96MM-S were inoculated intragastrically to Balb/c mice. GPU993-S and GPU96MM-S were resistant to streptomycin. Mice inoculated with these bacteria were given drinking water supplemented with streptomycin throughout the experimental period. MMC was injected i.p. at 18, 21 and 24 h, and anti-Stx-1 IgY or anti-Stx-2 IgY was given at 16, 19, 22 and 40 h after inoculation. Control mice were given PBS instead of anti-Stx IgY. Each group included 6 mice. There was a significant difference at *p<0.05 vs. the control (log rank test).

The effect of anti-Stx-1 IgY and anti-Stx-2 IgY on mortality of mice with intestinal infection with GPU993-S and GPU96MM-S (Table 1) was investigated (Figure 6). Mitomycin C (MMC) was injected i.p. at 18, 21 and 24 h after the bacterial inoculation to enhance Stx-2 production from the bacteria. IgY was administered orally at 16, 19, 22 and 40 h. After infection with GPU993-S (Figure 6A), three out of six mice in the control group and four out of six treated with anti-Stx-1 IgY (200 mg/kg) died, whereas none of the six mice treated with any dose of anti-Stx-2 IgY (50, 100 and 200 mg/kg) died. After infection with GPU96MM-S (Figure 6B), four out of six mice died in the control group. However, two out of six mice died after treatment with 20 mg/kg anti-Stx-2 IgY, and none of the six mice treated with 100 mg/kg anti-Stx-2 IgY.

### IgY concentration in feces of mice given anti-Stx-2 IgY orally

**Figure 7:**
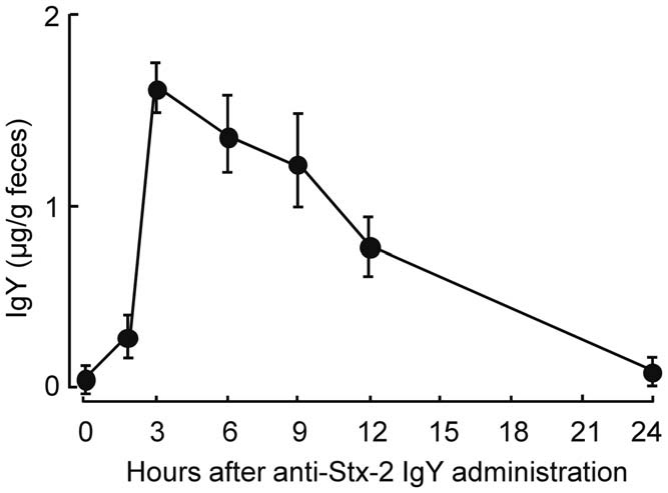
Time course of IgY concentration in feces of mice given anti-Stx-2 IgY orally. ddY mice were given 200 mg/kg anti-Stx-2 IgY orally. IgY concentration was measured by ELISA. Each point represents the mean ± SEM of five mice.

The time course of fecal IgY concentration was monitored in mice administered 200 mg/kg anti-Stx-2 IgY orally. IgY was detected in the feces collected at 2 h after administration, and reached a peak of 1.6±0.1 µg/g feces at 3 h, and then decreased to almost half the peak level at 12 h (Figure 7).

## Discussion

In this study, we immunized chickens with formalin-inactivated Stx-1 and Stx-2 and obtained anti-Stx-1 IgY and anti-Stx-2 IgY. It has been reported that rabbits immunized with Stx-1 or Stx-2 do not produce cross-neutralizing antibody [36], [38], [39]. First, we characterized the reactivity of antibodies in the IgY against Stx-1 and Stx-2. Western blot analysis indicated that the IgY reacted with the A subunit and A1 fragment of the corresponding toxin, but did not bind to the monomeric form of the B subunit. Visualization of the Stx B subunits by CBB staining suggested that a sufficient amount of antigen was present in the blotted lane for detection of any antibody reactive against monomeric B subunit.

By using SDS-PAGE, proteins were denatured and B subunits were converted from a polymeric form to monomers. Subsequently, the cross-reactivity of IgY against hpStx-1 and hpStx-2 was examined by dot blot assay. The results suggest that IgY was poorly cross reactive even when Stx had a native A subunit and polymeric B subunit. Interestingly, the IgY reacted with the recombinant B subunit, Stx-1BH and Stx-2BH, that has previously been reported to be in a polymeric form [40], even though the IgY did not react with the monomeric B subunit in the SDS-PAGE immunoblotting assay. These results indicated that anti-Stx-1 IgY and anti-Stx-2 IgY react with the polymeric form of the B subunit but not the monomeric form, with almost no cross-reactivity against the polymeric B subunit.

Neutralizing activity of anti-Stx-1 IgY and anti-Stx-2 IgY on Stx-1 and Stx-2 cytotoxicity in HeLa 229 cells was studied. Similar levels of Stx-1 and Stx-2 were required to induce cytotoxicity in HeLa 229 cells, which is in agreement with several reports [41]–[44]. However, there are also reports showing that Stx-1 is more toxic than Stx-2 [45], [46]. This discrepancy may be attributable to the number and type of glycolipid receptors present on the cells used in the experiments. The results indicated that there was no cross-neutralizing activity of anti-Stx-1 IgY and anti-Stx-2 IgY, as reported previously in rabbits [36], [38], [39]. To establish which of the antibodies against the Stx A subunit or polymeric B subunit contributes to neutralization of cytotoxicity, neutralizing activity of anti-Stx-1 IgY and anti-Stx-2 IgY was examined on Stx-2A1B. Anti-Stx-1 IgY neutralized Stx-2A1B efficiently but anti-Stx-2 IgY did not. In addition, the neutralizing activity of anti-Stx-1 IgY was suppressed by Stx-1BH but not Stx-2BH, and the activity of anti-Stx-2 IgY was suppressed by Stx-2BH but not Stx-1BH. These findings suggest that the antibody against the polymeric form of B subunit plays a major role in neutralizing cytotoxicity rather than the A subunit antibody against A subunit, although there is a report that mouse monoclonal antibodies against Stx-2 A subunit neutralize Stx-2 [27]. Moreover, anti-Stx-1 IgY and anti-Stx-2 IgY did not inhibit the cytotoxicity of bound Stx on the surface of target cells. Antibodies that recognize the conformational structure of the polymeric B subunit might inhibit binding of holotoxin through the B subunit to the receptor, such as Gb_3_-Cer, to exert their neutralizing activity. It is recently reported that IgY of chicken immunized with recombinant Stx-1 B subunit inhibited the binding of Stx-1 to target cells [47].

The preventive effect of anti-Stx-1 IgY and anti-Stx-2 IgY against death in mice injected i.p. with a lethal dose of Stx-1 or Stx-2 was investigated. Less toxicity was reported for Stx-1 compared with Stx-2 in mice [45], [48]. Here, ppStx-1 and ppStx-2 were injected i.p. at 625 µg/kg, corresponding to 50 µg/kg Stx-1, and 17.4 µg/kg corresponding to 1 µg/kg Stx-2, as a potentially lethal dose for almost all mice within a few days. Anti-Stx-1 IgY and anti-Stx-2 IgY showed protection following i.v. as well as i.p. administration in mice challenged i.p. with the corresponding Stx, without cross-preventive activity in agreement with the results *in vitro*. The experiment for time dependency of the treatment showed that anti-Stx-1 IgY and anti-Stx-2 IgY were effective when given 10 min before or simultaneously with Stx-1 and Stx-2, but not when given later than 10 min after Stx injection. Stx can enter the circulation within a few minutes after i.p. injection, and reach the target organs. It is reported that 90% of Stx-1 and Stx-2 is cleared from the blood within 5 min after i.v. injection in mice [49]. The results suggest that the antibodies in anti-Stx-1 IgY and anti-Stx-2 IgY cannot inhibit toxicity after binding of the toxins to target organs. This was supported by the *in vitro* experiment in which anti-Stx-1 IgY and anti-Stx-2 IgY did not neutralize Stx after binding to target cells. From the perspective of preventing Stx-mediated diseases, Stx should be inactivated in the intestine before it enters the circulation, rather than in the blood stream. However, there were reports describing that monoclonal antibody to Stx-2 A subunit prevented the death of piglets infected intestinally with EHEC even when the antibody was given i.p. 24 to 48 h after the EHEC infection [26], [50]. In this case, diarrhea started after 16 h and neurological symptoms at 48 to 96 h due to Stx-2 suggesting that Stx entered the bloodstream from the intestine after this point. The i.p. administration of the monoclonal antibody at 24 to 48 h may precede entering of Stx to the bloodstream in this piglet model.

Anti-Stx-1 IgY and anti-Stx-2 IgY were examined for oral efficacy to prevent the death of mice infected intestinally with EHEC O157:H7. GPU993-S produced only Stx-2, and GPU96MM-S produced Stx-1 and Stx-2. MMC treatment enhances Stx-2 production of EHEC in the intestine [51]. These experiments were designed to examine not only if the IgY inactivates Stxs in the intestine but also the potential of co-administration of the IgY with antibiotics simulating the likely clinical setting for EHEC infection, although MMC was used instead of antibiotics. Stx-2 concentration in the cecum increases drastically 3 h after the first MMC treatment and peaks 6 h after (HM un-published data). The IgY is expected to reach the colon 2 to 3 h after oral administration as supported by the data in Figure 7. Then, anti-Stx-1 IgY and anti-Stx-2 IgY were given orally at the time of MMC treatment. Anti-Stx-2 IgY protected mice from death caused by GPU993-S infection, but anti-Stx-1 IgY did not affect mortality. Anti-Stx-2 IgY also prevented death of mice infected with GPU96MM-S. It is likely that anti-Stx-2 IgY suppressed entry of Stx-2 into the circulation, which resulted in prevention of death. IgY given orally was detected in the feces by a peak concentration at 3 h after administration. It is expected that oral IgY reaches the large intestine to express its antibody activity. The mechanism for Stx to enter the circulation from the intestine is not precisely known, although there are several reports about the mechanisms 52–54. It is still possible that antibody against Stx A subunit contributes to suppression of Stx entry into the circulation, while antibody against A subunit is not involved in neutralization of Stx, as shown in this study. It remains to be established which antibodies against A subunit and B subunit are effective for inactivation of Stx in the intestine to elucidate the precise mechanism for the IgY to inactivate Stx in the intestine.

The oral efficacy of anti-Stx-1 IgY has not yet been determined in an intestinal infection model in mice, because we have no model in which Stx-1 is produced in the intestine and causes death. This also remains to be studied, although Stx-2 is more toxic than Stx-1 in mice [48], and more likely to be involved in the development of HUS in humans [20], [55].

In conclusion, we demonstrated that chickens immunized with Stx toxoid can produce IgY antibodies that can inactivate Stx in the intestine. It is possible to obtain IgY from egg yolk in large amounts at low cost. Anti-Stx-1 IgY and anti-Stx-2 IgY have the potential to be used clinically for the prevention of Stx-mediated diseases. It is possible to use oral IgY in combination with antibiotics to help reduce the risk of Stx-mediated diseases due to the use of antibiotics.

## Materials and Methods

### Bacterial strains

EHEC GPU96MM was a clinical isolate of EHEC O157:H7, which produced Stx-1 and Stx-2 in the outbreak at Gifu, Japan in 1996 [56]. *E. coli* BL21/pETSTX1 was a recombinant strain that produced Stx-1 [17]. *E. coli* GPU993 was a *stx1*-deletion mutant of EHEC GPU96MM that produced Stx-2 [57]. *E. coli* JM109/p2Ais-10 [58] was a recombinant strain that produced a hybrid toxin composed of A subunit of Stx-2 and B subunit of Stx-1 (Stx-2A1B).

Resistance to streptomycin was spontaneously induced in GPU96MM and GPU993 by passaging in nutrient broth (Nissui Pharmaceutical, Tokyo, Japan) that contained 5 mg/ml streptomycin sulfate (Meiji Seika, Tokyo, Japan), and defined as GPU96MM-S and GPU993-S, respectively, to be used for the infection experiments.

### Experimental animals

Commercially available chickens were maintained under conventional conditions in the animal room of the Immunology Research Institute, GHEN Corporation (Gifu, Japan), and immunized with Stx-1 and Stx-2 at 24 weeks of age. The experiments were performed in accordance with the Guidance for the Care and Use of Laboratory Animals of GHEN Corporation in Nov. 2004. All procedures that involved animals were approved by the Animal Care and Use Committee of Immunology Research Institute, GHEN Corporation (approval number 04-1128).

SPF female ddY mice and SPF female Balb/c mice were obtained at 4 weeks of age from Japan SLC (Hamamatsu, Japan), and used for the experiments after 1 week acclimation. Mice were maintained in the Laboratory for Animal Experiments of Gifu Pharmaceutical University (Gifu, Japan), with free access to sterilized rodent chow (CRF-1; Oriental Yeast, Tokyo, Japan) and water. The experimental designs and all procedures were approved by the Animal Experiment Committee of Gifu Pharmaceutical University (permission number; 05-130, 05-154, 05-363, 06-082, and 06-132). All procedures relating to animal care and treatment conformed to animal care guidelines of this committee.

### Partial purification of Stx-1, Stx-2 and Stx-2A1B

*E. coli* BL21/pETSTX1 was grown at 37°C with shaking in Luria–Bertani broth supplemented with 60 µg/l ampicillin (Wako Pure Chemicals Industries, Osaka, Japan) to prepare Stx-1. At the mid-logarithmic phase, isopropyl β-D(-)- thiogalactopyranoside (Wako Pure Chemicals Industries) was added at a final concentration of 1 mM, and the culture was continued for an additional 6 h. The cell pellet harvested by centrifugation was resuspended in PBS (pH 7.4) that contained polymyxin B (0.1 mg/ml), incubated at 37°C for 30 min, and then centrifuged. The supernatant was filtered through a 0.22-µm filter (Millex; Millipore). The filtrate was then extensively dialyzed and concentrated against PBS using Stirred Ultrafiltration Cell with a 30 000 molecular weight cut-off membrane (Amicon; Millipore). The preparation was referred to as partially purified Stx-1 (ppStx-1). Partially purified Stx-2A1B (ppStx-2A1B) was prepared from *E. coli* JM109/p2Ais-10 in the same method described above for ppStx-1.

*E. coli* GPU993 was cultured at 37°C with shaking in modified Syncase medium [17] to prepare Stx-2. At the mid-logarithmic phase of growth, 0.6 µg/ml MMC (Kyowa Hakko Kogyo, Tokyo, Japan) was added to the culture to stimulate Stx-2 production, followed by cultivation for an additional 6 h. The culture supernatant obtained by centrifugation was concentrated by ultrafiltration with 30 000 molecular weight cut-off membrane (Pellicon 2 Cassette filter Biomax 30; Millipore). The filtrate was extensively dialyzed and concentrated against PBS using Stirred Ultrafiltration Cell with a 30 000 molecular weight cut-off membrane (Amicon, Millipore). This preparation was referred to as partially purified Stx-2 (ppStx-2).

Stx-1 and Stx-2 concentrations and cytotoxic activities were assayed by ELISA and cytotoxicity assay described below. The Stx-1 concentration in ppStx-1 was 80.1 µg/mg protein and Stx-2 in ppStx-2 was 57.6 µg /mg protein. The CD_50_ against HeLa 229 cells was 8.8 pg/ml Stx-1 in ppStx-1 and 1.1 pg/ml Stx-2 in ppStx-2.

### Highly purification of Stx-1, Stx-2 and Stx-2A1B

Stx-1, Stx-2 and Stx-2A1B were highly purified from the respective partially purified preparations (ppStx-1, ppstx-2 and ppStx-2A1B) by affinity chromatography using Gb_3_-Cer (Nacalai Tesque) as described previously [17], [59], and referred to as hpStx-1, hpStx-2 and hpStx-2A1B, respectively. The CD_50_ was 9.3 pg/ml, 1.2 pg/ml and 8.2 pg/ml in hpStx-1, hpStx-2 and hpStx-2A1B, respectively.

### Purification of recombinant B subunit of Stx-1 and Stx-2

Stx-1BH and Stx-2BH were purified by affinity chromatography with Ni^2+^-loaded HiTrap Chelating HP column (Amersham Biosciences, Tokyo, Japan) as described previously [40], [60], and referred to as Stx-1BH and Stx-2BH, respectively.

### Immunization of chickens with ppStx-1 and ppStx-2

ppStx-1 and ppStx-2 were diluted with PBS, mixed with 0.5% final concentration formalin, and kept for 3 days at 37°C. The formalin-inactivated preparations were mixed with an equal volume of Freund's complete adjuvant (Difco, Detroit, MI, USA), and the mixture was injected into two different sites of the breast muscle (0.5 ml per site, 50 µg Stx-1 or Stx-2/chicken). One month later, the chickens were boosted by injecting the inactivated preparation with Freund's incomplete adjuvant (Difco) in the same manner (100 µg Stx-1 or Stx-2/chicken). Two chickens were used for each immunization with Stx-1 and Stx-2.

### Purification of anti-Stx-1 and anti-Stx-2 IgY

The eggs were harvested at 2–7 weeks after the booster, and the yolks were isolated and pooled. The pooled yolk was well mixed with 10 times volume of distilled water, and kept at 4°C overnight. Ammonium sulfate was added to the supernatant to bring the saturation to 40%. The overnight precipitate was dissolved with 40 mM NaCl, and then dialyzed against a sufficient quantity of 40 mM NaCl. The dialyzed solution was freeze-dried.

### ELISA to detect Stxs

Stx-1 and Stx-2 concentrations were determined by ELISA [61] using mouse anti-Stx-1B subunit monoclonal antibody (STX1-13C4; Toxin Technology, Sarasota, FL, USA) or anti-Stx-2B subunit monoclonal antibody (STX2-BB12; Toxin Technology) as a capture antibody, and mouse anti Stx-1 and Stx-2 pooled antibodies conjugated to horseradish peroxidase (STXPC-1; Toxin Technology) as a developing antibody. Purified Stx-1 and Stx-2 purchased from Nacalai Tesque, Inc. (Kyoto, Japan) were used to prepare the standard curves.

### Cytotoxicity assay to detect Stx-neutralizing activity

The cytotoxicity assay was performed as described previously [17], [18]. Briefly, 5×10^3^/well of HeLa 229 cells (kindly provided by Dr. T. Yoshida, Aichi Medical University, Aichi Prefecture, Japan) were cultured in Dulbecco's Modified Eagle's Medium (DMEM) (Nissui Pharmaceutical) supplemented with 10% heat-inactivated fetal bovine serum (ICN Biomedicals, Aurora, OH, USA), 100 U/ml penicillin G and 0.1 mg/ml streptomycin with 96-well flat bottomed culture plates (Costar; Corning Inc., Corning, NY, USA). After 24 h incubation, serial dilutions of hpStx-1 or hpStx-2 were added to the wells. Then, the cultures were continued for 48 h. AlamarBlue (Trek Diagnostic Systems, East Grinstead, West Sussex, UK) was added to the wells, followed 2 h later by measuring fluorescence intensity (FI) at 590 nm (530 nm excitation, CytoFluor 2350; Millipore, Bedford, MA, USA) to estimate the number of viable cells. The CD_50_ of Stx-1 and Stx-2 was estimated from the dose–response curves of the serial dilutions.

To evaluate Stx neutralizing activity of anti-Stx-1 and anti-Stx-2 IgY, the appropriately diluted IgY were mixed with five times CD_50_ hpStx-1 or five times CD_50_ hpStx-2, incubated at 37°C for 1 h, and then subjected to the cytotoxicity assay. Neutralizing concentration of the IgY was calculated as follows and the NC_50_ was calculated:
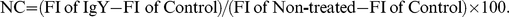



### Western blotting of anti-Stx-1 and anti-Stx-2 IgY

hpStx-1 and hpStx-2 (2.5 µg/lane) were subjected to SDS-PAGE (15% polyacrylamide gel). The gels were stained with CBB R-250. For western blotting, proteins were transferred to 0.22-µm polyvinylidene difluoride membranes (Nippon Genetics, Tokyo, Japan) with a semi-dry transfer apparatus (BE-330; Bio Craft, Tokyo, Japan). The membrane was blocked for 1 h with PBS that contained 1% bovine serum albumin, 5% normal calf serum and 0.1% Tween 20 (BB). Anti-Stx-1 IgY and anti-Stx-2 IgY, diluted to 20 µg/ml in BB, were applied to the membranes and incubated for 5 h. The membranes were then incubated for 2 h with rabbit anti-chicken IgG conjugated to horseradish peroxidase (Zymed, San Francisco, CA, USA) diluted at 1∶4000 in BB. The reaction was visualized by a substrate solution of 0.5 M Tris–HCl buffer (pH 6.8) that contained 0.2 mg/ml 3, 3′-diaminobenzidine tetrahydrochloride (Wako Pure Chemicals Industries) and 0.2 µl/ml 30% H_2_O_2_.

### Dot blot analysis of anti-Stx-1 and anti-Stx-2 IgY

hpStx-1 and hpStx-2 were blotted onto 0.22-µm polyvinylidene difluoride membranes (Nippon Genetics) at 0.4 µg/10 µl/dot, and Stx-1BH and Stx-2BH at 2 µg/10 µl/dot. Anti-Stx-1 IgY (2 µg/ml) and anti-Stx-2 IgY (4 µg/ml) diluted in BB were applied to the dot and incubated for 5 h. The following procedure to stain was the same as described for western blotting.

### Mouse mortality assay by i.p. challenge with ppStx-1 and ppStx-2

ddY mice were used for the experiment. The 50% lethal dose (LD_50_) of ppStx-1 was 250 µg/kg (20 µg/kg as Stx-1) and that of ppStx-2 was 8.7 µg/kg (0.5 µg/kg as Stx-2). In the mortality assay to examine the *in vivo* neutralizing activity of anti-Stx-1 IgY and anti-Stx-2 IgY, mice were challenged i.p. with 625 µg/kg ppStx-1 (2.5 times LD_50_) and/or 17.4 µg /kg ppStx-2 (two times LD_50_). Mouse survival was observed for 10 days after the challenge.

### Intestinal infection with EHEC O157:H7

The infection experiment with GPU993-S and GPU96MM-S was performed as described previously [17]. Balb/c mice were given drinking water supplemented with 5 mg/kg streptomycin, continuously from 2 days before infection throughout the experimental period. Mice were starved of food for 8 h and then injected i.p. with 25 mg/kg cimetidine, followed 15 min later by intragastric inoculation with 5×10^4^ cfu/kg GPU993-S or GPU96MM-S. MMC was injected i.p. at a dose of 0.25 mg/kg at 18, 21 and 24 h after bacterial inoculation. Mouse survival was observed for 14 days after the inoculation.

### Measurement of IgY concentration in feces

ELISA was used to measure IgY concentration in feces of mice given anti-Stx-2 IgY orally. Two or more fecal pellets were collected from the mice, weighed, and suspended in five volumes of PBS that contained 0.1% sodium azide. The pellets were crushed by shaking vigorously in a tube that contained glass beads. The suspension was then centrifuged at 15 000 rpm for 20 min to obtain a clear supernatant. In the ELISA to measure the IgY concentration in the supernatant, rabbit anti-chicken IgG (Bethyl, Montgomery, TX, USA) was used as a capture antibody and rabbit anti-chicken IgG conjugated to horseradish peroxidase (Zymed) as a developing antibody. Commercially available purified chicken IgG (Cappel, Aurora, OH, USA) was used to make a standard curve. IgY concentration (µg/g feces) was calculated from the curve using GraphPad PRISM (GraphPad Software, San Diego, CA, USA).

### Statistical analysis

Results are presented as the mean ± SEM. GraphPad PRISM (GraphPad Software) was used to calculate CD_50_ and NC_50_, and statistical analyses. Neutralizing activity of IgY preparations was evaluated by Dunnet's comparison test after one way analysis of variance (Figures 2–4). Survival time of mice was evaluated by log rank test (Tables 3 and 4, Figures 5 and 6). A value of *p*<0.05 was considered to be statistically significant in all analyses.
